# The Evolution of the *KANADI* Gene Family and Leaf Development in Lycophytes and Ferns

**DOI:** 10.3390/plants8090313

**Published:** 2019-08-30

**Authors:** Cecilia Zumajo-Cardona, Alejandra Vasco, Barbara A. Ambrose

**Affiliations:** 1 The New York Botanical Garden, Bronx, NY 10458, USA; 2 Biology department, The Graduate Center, City University of New York, New York, NY 10016, USA; 3 Botanical Research Institute of Texas, Fort Worth, TX 76107, USA

**Keywords:** *Equisetum*, ferns, in situ hybridization, lycophytes, *KANADI*, megaphyll, microphyll, plant evo-devo, *Selaginella*, telome theory

## Abstract

Leaves constitute the main photosynthetic plant organ and even though their importance is not debated, the origin and development of leaves still is. The leaf developmental network has been elucidated for angiosperms, from genes controlling leaf initiation, to leaf polarity and shape. There are four *KANADI* (*KAN*) paralogs in *Arabidopsis* *thaliana* needed for organ polarity with *KAN1* and *KAN2* specifying abaxial leaf identity. Yet, studies of this gene lineage outside angiosperms are required to better understand the evolutionary patterns of leaf development and the role of *KAN* homologs. We studied the evolution of *KAN* genes across vascular plants and their expression by in situ hybridization in the fern, *Equisetum hyemale* and the lycophyte *Selaginella moellendorffii*. Our results show that the expression of *KAN* genes in leaves is similar between ferns and angiosperms. However, the expression patterns observed in the lycophyte *S. moellendorffii* are significantly different compared to all other vascular plants, suggesting that the *KAN* function in leaf polarity is likely only conserved across ferns, gymnosperms, and angiosperms. This study indicates that mechanisms for leaf development are different in lycophytes compared to other vascular plants.

## 1. Introduction

Leaves are the most easily recognizable plant organ, yet surprisingly they may have evolved independently more than once [1]. Leaves are thought to have evolved between 2 and 11 times in vascular plants (lycophytes, ferns, and seed plants). Leaves in lycophytes are commonly termed microphylls while leaves in ferns and seed plants are termed megaphylls [1,2,3,4]. The fossil record indicates that leaves evolved independently in lycophytes and euphyllophytes (ferns and seed plants), and molecular genetics generally supports this idea [2,3,5,6,7,8]. The discrepancy in the number of times leaves have evolved is due to varying results mainly within ferns due to an incomplete fossil record and the paucity of developmental genetic studies in this group [1]. The most widely accepted theory for leaf evolution is the telome theory where leaves are proposed to have evolved through independent processes of branching, overtopping, planation, and webbing [9]. The evolution of webbing would result in the development of a laminar structure.

**Figure 1:**
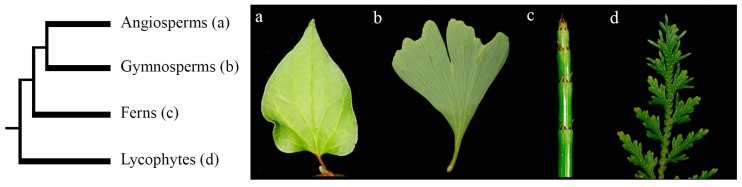
Schematic representation of the evolution of vascular plants and examples of their leaves. (**a**) Angiosperm *Houttuynia cordata* single simple leaf; (**b**) gymnosperm *Ginkgo biloba* single simple leaf; (**c**) fern *Equisetum hyemale* shoot with whorls of leaves; (**d**) lycophyte *Selaginella moellendorffii* shoot with leaves.

Leaves are usually thought of as the flat lateral organs in the plant, because one of the main characters that defines most leaves is their bilateral symmetry. As the leaf primordium develops, it generally forms two anatomically distinct surfaces, the adaxial and abaxial sides. Morphological diversity of leaves across land plants can be associated, in many instances, to changes in the adaxial/abaxial identity (Figure 1) [10]. Experimental evidence indicates that the specification and maintenance of bilateral symmetry during leaf development is necessary for lamina outgrowth [10,11,12,13].

The genetic network specifying the proper development of axes during leaf development and consequently lamina outgrowth, particularly in *Arabidopsis thaliana*, has been well studied and involves multiple regulatory mechanisms, from transcription factors to small RNAs and hormones [10,11,12,13]. Briefly, *ASYMMETRIC LEAVES1* and *2 (AS1/AS2)* [14,15,16,17], along with small RNAs, regulate *Class III HD-Zips,* which specify adaxial identity in *A. thaliana* [13,15,16,17]. While abaxial identity of the *A. thaliana* leaf, is specified by *ETTIN/AUXIN RESPONSE FACTOR 3(ETT/ARF3)*, *ARF4,* and *KANADIs (KANs)* [18,19,20,21,22,23,24,25,26,27,28]. Of particular importance is the mutual antagonism of the specification of adaxial and abaxial leaf identities [29]. That is, in mutants lacking adaxial identity, the organ has abaxial identity only, and vice versa [12,13,18,19,20]. In addition, if adaxial or abaxial identity is lost then the resulting organ is more radial rather than a flat laminar structure [11,12,13].

Comparative studies to understand the specification of ab/adaxial identity have mainly focused on angiosperms [reviewed in 10], with the exception of expression studies of *AS1* homologs in the lycophyte *Selaginella kraussiana* and the fern *Osmunda regalis* [6], and *Class III HD-Zip* expression studies in lycophytes and ferns [8,30,31]. Similar to results found in seed plants;,*Class III HD-Zips* have been shown to be expressed in the adaxial side of leaf primordia throughout ferns from *Equisetum* to leptosporangiat*e* ferns [8]. However, expression studies of *Class III HD-Zips* in lycophytes, show that these genes are specifically expressed in leaf primordia, but the expression is not polar and is not maintained in older leaves [8,30,31]. These results suggest that the function of *Class III HD-Zips* genes in leaf adaxial identity is only conserved between ferns and seed plants but not lycophytes.

To better understand the evolution and development of bilateral symmetry in vascular plant leaves, we investigated *KANADI* homologs across lycophytes and ferns. *KAN* genes belong to the *GARP* family of transcription factors, characterized by the plant-specific *GARP* DNA binding domain. This domain is part of the helix-loop-helix distantly related to the *MYB* domain [27]. The *GARP* domain is crucial for regulating the transcription of downstream genes [19]. There are four *KAN* paralogs in *Arabidopsis thaliana (KAN1–4),* even though *KAN1* and *KAN2* have been described by their function in leaf polarity, all four *KAN* paralogs are involved in proper determination of organ polarity, including the integuments of the ovules [19,32,33,34]. Phylogenetic analyses for the *KANADI* gene family have been focused on angiosperm model species, including *Arabidopsis thaliana*, *Zea mays*, and *Oryza sativa* homologs [27]. Functional studies have also been performed only in these three model species, and indicate conserved functions in the proper determination of the abaxial leaf identity [27,28].

Available data regarding the evolution and expression of *KAN* genes are not sufficient to assess how these genes have functionally changed over time and their implications in leaf morphological evolution. Here we provide the first phylogenetic tree with sampling across vascular plants, with a focus on lycophytes and ferns. Furthermore, we present the expression of *KANADI* homologs in the developing shoots of the lycophyte *Selaginella moellendorffii* and the fern *Equisetum hyemale,* that allow us to hypothesize about the functional evolution of the *KAN* genes and the leaf developmental network in vascular plants.

## 2. Materials and Methods

### 2.1. KANADI Phylogenetic Analyses across Vascular Plants

We used the Basic Local Alignment Search Tool (BLAST) with the four *KANADI* paralogs, in nucleotides, from *Arabidopsis thaliana* (At5g16560, At1g32240, At4g17695, and At5g42630) as queries. We focused mainly on fern sequences publicly available in the OneKP transcriptome database (https://sites.google.com/a/ualberta.ca/onekp/) [35,36,37] with 28 sequences included; 25 sequences from gymnosperms and angiosperms available in the genome database Phytozome (https://phytozome.jgi. doe.gov/pz/portal.html) [38] and the NCBI (https://www.ncbi.nlm.nih.gov/; Table S1). Additionally, we included *Ceratopteris richardii* transcriptome sequences from the Ceratopteris Genome Project. Sequences were aligned with the online version of MAFFT [39] (https://mat.cbrc.jp/alignment/server/) with a gap open penalty of 3.0, an offset value of 0.5, and all the other parameters by default. Alignment was manually refined with AliView [40]. Phylogenetic relationships were inferred by Maximum Likelihood (ML) analysis using the complete nucleotide alignment, through the CIPRES Science Gateway [41] with RaxML-HPC BlackBlox [42]. The final topology was observed and edited using FigTree v.1.4.3 [43]. This analysis included, as ingroup, 53 sequences from across vascular plants, and three *KANADI* sequences from *Physcomitrella patens* were used as outgroups (Table S1).

### 2.2. In Situ Hybridization Expression Analyses for KANADI Homologs in Ferns and Lycophytes

*Equisetum hyemale* and *Selaginella moellendorffii* plants were grown under controlled conditions in the NYBG Nolen greenhouses. Young shoots from *Equisetum hyemale,* and sterile and fertile shoots from *Selaginella moellendorffii* were collected and immediately fixed in formaldehyde-acetic acid-ethanol and water (FAA; 3.7% formaldehyde: 5% glacial acetic acid: 50% ethanol). The material was dehydrated through an alcohol-Histo-Clear II (National Diagnostics, Atlanta, GA, United States) series and embedded in Paraplast X-tra (Fisher Healthcare, Houston, TX, United States). The samples were sectioned at 8 μm with a MICROM HM355 (Fisher Scientific, Pittsburgh, PA, United States) rotary microtome. DNA templates for RNA probe synthesis were obtained by amplification of 290–350 bp fragments. To ensure specificity, the probes were designed flanking the *GARP* domain (Table S2) *S. moellendorffii* primers were designed towards the 3’ end of the sequence (Figure S1) while *E. hyemale* primers were designed towards the 5’ UTR end of the sequence. Fragments were cleaned using QIAquick PCR purification Kit (Qiagen, Valencia, CA, United States). Digoxigenin labeled RNA probes were prepared using T7 RNA polymerase (Roche, Switzerland), RNAse inhibitor RNasin (New England Biolabs, Ipswich, MA, United States), and RNA labeling-mix (Roche, Switzerland) according to the manufacturer’s protocol. RNA in situ hybridization was performed according to Ambrose et al. (2000) [44].

## 3. Results

### 3.1. Evolution of KANADI in Lycophytes and Ferns

Phylogenetic analyses include representatives from all major plant lineages across vascular plants containing lycophytes, ferns, gymnosperms, and angiosperms, for an in-group of 53 sequences and three *Physcomitrella* homologs used as outgroups (Table S1). Angiosperm sequences are for the most part complete coding sequences as they were downloaded from genome databases, whereas some gymnosperm and fern sequences are missing 30–60 amino acids (AA) from the start codon, and others lack the stop codon. We were able to identify the *GARP* domain in all the sequences included in the analysis.

**Figure 2:**
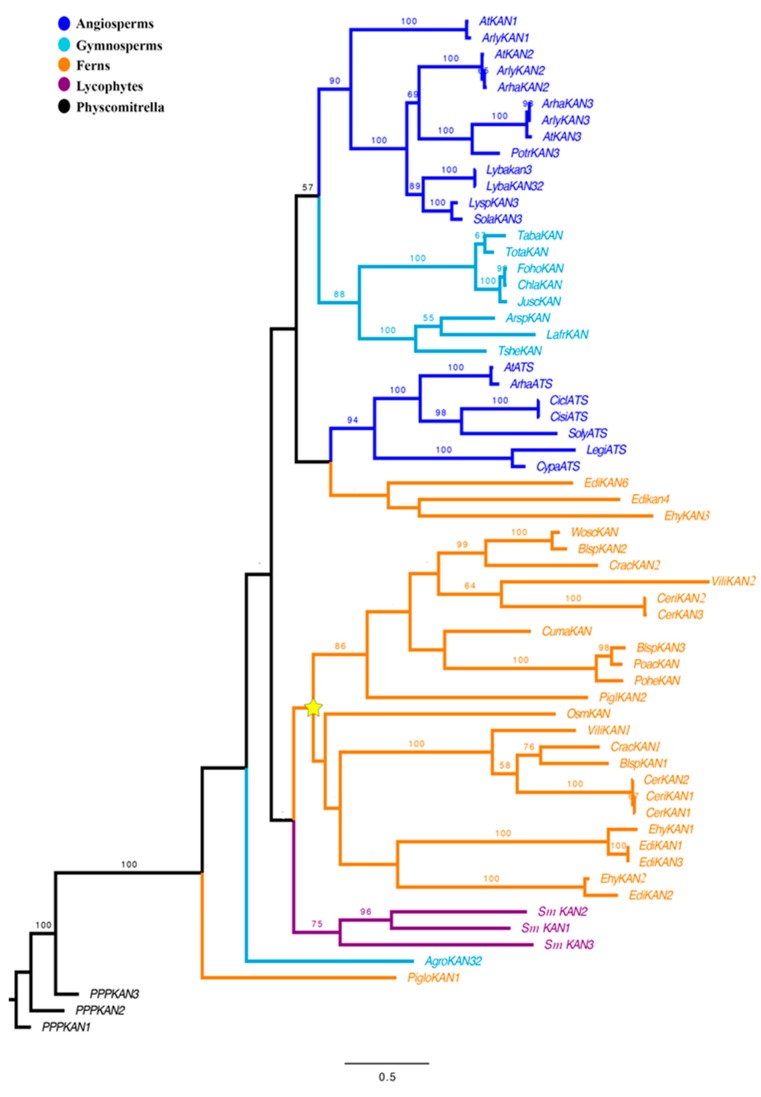
A maximum likelihood hypothesis for the evolution of *KANADI* genes across vascular plants. The key to the colors in the topology is shown in the upper left corner of the figure. The yellow star indicates a possible duplication event within ferns. Bootstrap (BS) values higher than 50 are shown on the corresponding branch.

Our maximum likelihood (ML) analyses recovered two clades of fern *KAN* homologs, one sister to *Arabidopsis* ATS comprised only by *Equisetum* sequences, *EdiKAN4*, *6* and *EhyKAN3* and the other, sister to all lycophyte sequences (Figure 2). The clade containing most fern sequences has two subclades, likely as the result of a major duplication event (Figure 2). Both subclades include sequences from several ferns such as *Blechnum spicant*, *Ceratopteris richardii*, *Ceratopteris thalictroides*, *Cryptogramma acrostichoides*, and *Vittaria lineata* (Figure 2). Equisetum sequences are recovered in only one of the subclades, thus it is unclear if the inferred duplication occurred before or after the evolution of *Equisetaceae* (Figure 2). Sister to these two fern clades are the three *S. moellendorfii* homologs included in this study, possibly as the result of a limited sampling among lycophytes (Figure 2).

### 3.2. Expression of KANADI in the Lycophyte Selaginella moellendorffii

**Figure 3:**
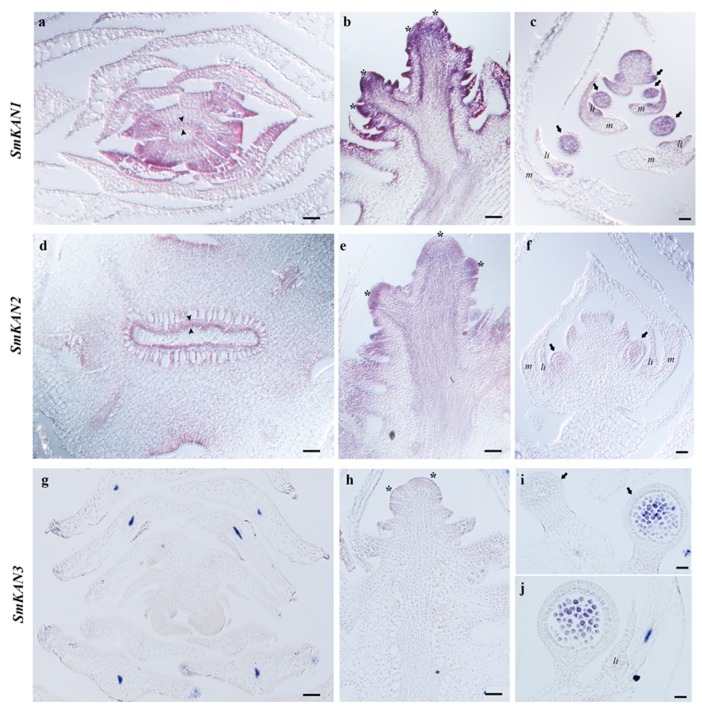
*SmKAN* expression in *Selaginella moellendorffii* by in situ hybridization. (**a**–**c**) *SmKAN1* expression patterns. (**a**) *SmKAN1* expression is detected in the vasculature in a transverse section of a shoot. (**b**) *SmKAN1* expression is detected in the emerging microphyll primordia but not the meristem of a longitudinal section through a shoot. (**c**) *SmKAN1* expression is detected in the emerging sporangia primordia and is maintained throughout their development in a longitudinal section through a strobilus. (**d**–**f**) *SmKAN2* expression patterns. (**d**) *SmKAN2* expression is detected in the vasculature in a transverse section of shoot. (**e**) *SmKAN2* expression is detected in the emerging microphyll primordia but not the meristem of a longitudinal section through a shoot. (**f**) *SmKAN2* expression is detected in the emerging sporangia primordia and is maintained throughout their development in a longitudinal section through a strobilus. (**g**–**j**) *SmKAN3* expression results. (**g**) Cross section of a shoot, *SmKAN3* expression is not detected. (**h**) Expression of *SmKAN3* is not detected in the leaf primordia. (**i**) *SmKAN3* expression is not detected during early sporangia development but is detected late in sporocoyte proliferation. (**j**) *SmKAN3* expression in a nearly mature sporangium prior to meiosis. Scale bars: 50 µm (**a**,**b**,**e**,**g**); 20 µm (**c**,**d**,**f**,**h**); 10 µm (**i**,**j**). *Asterisk* indicates apical meristem; *black arrowheads* indicate phloem; *black arrows* indicate sporangia; li = ligule; m = microphyll.

To better understand the role of *KANADI* homologs in lycophytes, we examined the expression of these genes in the heterosporous lycophyte *Selaginella moellendorffii* in vegetative and reproductive tissues by in situ hybridization (Figure 3).

We found the expression of *SmKAN1* and *SmKAN2* to be very similar except that *SmKAN1* appeared to be more highly expressed than *SmKAN2* at all developmental stages (Figure 3). In transverse sections of the shoot axis, *SmKAN1* expression is detected as a ring in the developing vasculature which likely represents the developing phloem (Figure 3a, arrowheads). Later in shoot development, the expression of *SmKAN2* is still detected in the developing vasculature, specifically in the phloem that surrounds the central xylem pole (Figure 3d). In longitudinal sections of the shoot axis, *SmKAN1* and *SmKAN2* are detected in the earliest emerging leaf primordia and as the leaf primordia continue to grow (Figure 3b,e). *SmKAN1* and *SmKAN2* expression is not detected in older leaves (Figure 3a,b,e). *SmKAN3* had a more discrete pattern of expression compared to *SmKAN1* and *SmKAN2*. Notably, *SmKAN3* expression is not detected during any stage of leaf development (Figure 3a,d,g). While expression of *SmKAN1* and *SmKAN2* during *S. moellendorfii* development is detected in the leaf primordia, neither of them show expression patterns polarized to one side of the developing leaf (Figure 3a,b,e,h). In addition, *SmKAN1*, *SmKAN2*, and *SmKAN3* expression is not detected in the shoot apical meristem (SAM).

During development of the *S. moellendorffii* strobilus (fertile sporophytic axis), the expression of *SmKAN1* and *SmKAN2* was analogous to what was found in the vegetative shoot axis (Figure 3c,f). *SmKAN1*, *SmKAN2*, and *SmKAN3* expression is not detected in the meristem of the strobilus. However, *SmKAN1* and *SmKAN2* expression was found in the sporangium incipient primordia on the flanks of the strobilus and this expression is maintained in the sporangia at least through the proliferation of the sporocytes. Expression of *SmKAN3* is detected in sporangium development during later stages of sporocyte proliferation prior to meiosis (Figure 3i,j).

### 3.3. Expression of KANADI in the Fern Equisetum hyemale

**Figure 4:**
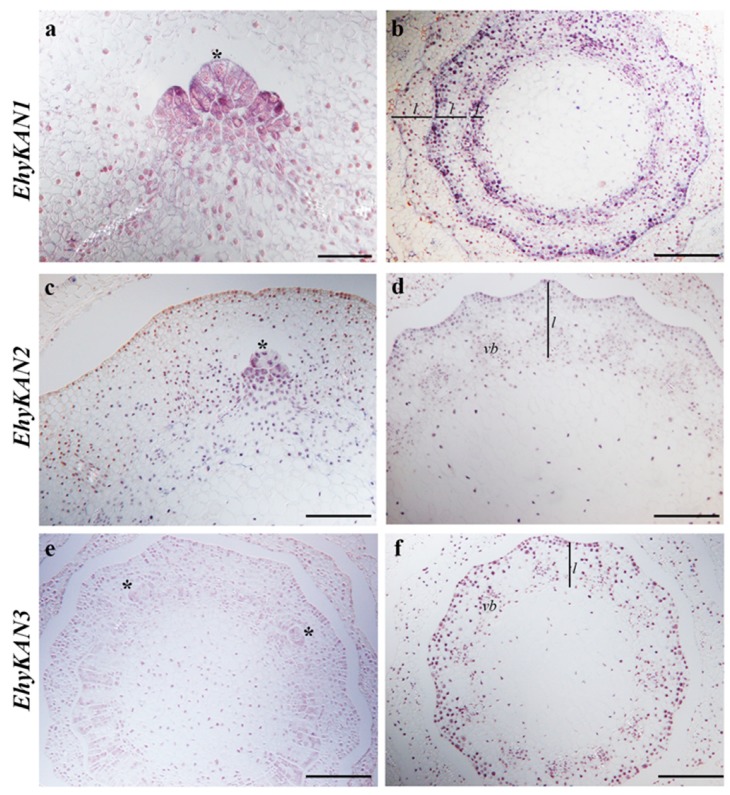
*EhyKAN* expression in *Equisetum hyemale* by in situ hybridization. (**a**) *EhyKAN1* expression is detected in the apical meristem and emerging leaf primordia of a branch in a transverse section of a shoot. (**b**) *EhyKAN1* expression is detected throughout the leaf primordia (innermost whorl) and then becomes restricted to the abaxial side of each leaf whorl (outer two whorls shown here) in a transverse section through a shoot. (**c**) *EhyKAN2* expression is detected in the apical meristem and emerging leaf primordia of a branch in a transverse section of a shoot. (**d**) *EhyKAN2* becomes restricted to the abaxial side of the leaf whorl in transverse section through a shoot. (**e**) *EhyKAN3* expression is detected in the apical meristem of a branch in a transverse section of a shoot. (**f**) *EhyKAN3* becomes restricted to the abaxial side of the leaf whorl in transverse section through a shoot. Scale bars: 50 µm (**a**); 100 µm (**b**–**f**). *Asterisk* indicates apical meristem; lines with *l* indicates leaf whorl; vb = vascular bundle.

To better understand the expression of *KANADI* homologs in ferns we assessed the expression pattern of these in the fern *Equisetum hyemale* (Figure 4).

The leaves of *Equisetum* are morphologically distinct from the more common fern leaves that arise with circinate vernation [1]. The leaves of *E. hyemale* are formed in a whorl around the shoot axis (Figure 1c) and are easily seen in transverse sections of the shoot (Figure 4b,d,f). In addition, branches do not form consistently at every node of *E. hyemale* as in other *Equisetum* species, but branches do occasionally form (Figure 4a,c,e). We found the expression of all three *KANADI* homologs similar to each other with only an apparent difference in the level of expression between copies. *EhyKAN1/2/3* are all expressed in the SAM (asterisk) and emerging leaf primordia (Figure 4a,c,e). However, *EhyKAN3* showed lower levels of expression in the shoot apical meristem compared with the other two homologs. Expression of *EhyKAN1*, *2*, and *3* is detected in vascular bundles (Figure 4b,d,f). Notably, we detected that the three homologs are expressed in the leaves early in the development and become restricted on the abaxial side as the leaves mature (Figure 4b,d,f).

## 4. Discussion

*KANADI* genes belong to the GARP family of transcription factors [45] and the *GARP* domain confers the DNA-binding function [45,46,47]. Our analysis focused strictly on determining the evolutionary history of *KANADI* homologs across vascular plants, lycophytes, ferns, and seed plants (Figure 2). By doing the alignment of *KAN* homologs, our results indicate that all *KAN* homologs have a DNA binding function, as the *GARP* domain is highly conserved across vascular plants. Phylogenetic analyses showing the evolutionary history of the *KANADI* gene lineage are scarce, most of them including only model species [24,27,28]. Therefore, we performed a BLAST search across all major plant groups (Figure 2). Even though the analysis was focused in understanding the evolution of the KAN genes in lycophytes and ferns, our results allow us to hypothesize that lycophyte and fern homologs appeared before the diversification of the traditionally known *KAN1*, *KAN2, KAN3* and *KAN4* (ATS) in seed plants. To determine at which point during the evolution of seed plants the four copies of *KANADIs* evolved, a more exhaustive search of homologs across seed plants will be needed. However, such duplications are most likely associated with a whole genome duplication event (WGD) [48,49,50]. We performed a search in publicly available databases for *KAN* homologs across ferns. Here we report a duplication event within one of the *KANADI* clades of ferns for the first time (Figure 2). This duplication could have been the result of the whole genome duplication predating the core leptosporangiate ferns [51]. Nevertheless, additional sequences are required in order to corroborate this hypothesis.

We examined the expression of *KANADI* homologs for the first time outside of model angiosperm species. We found similar expression patterns in the three *KAN* homologs found in *Equisetum hyemale*; all have polar expression, specifically in the abaxial side of developing leaves, suggesting that the three copies are redundant for leaf development (Figure 4). In order to assess differences in the expression of *E. hyemale* homologs, it would require looking at different tissues such as the strobili and the roots, as expression in these structures has been reported in angiosperms [52]. On the other hand, we have not detected polar expression of *KANADI* genes as expected in lycophytes. Significantly, the expression of *SmKAN1*, *SmKAN2*, and *SmKAN3,* is not detected in a polar fashion, namely the abaxial side of developing leaves (Figure 3).

Our results for the *KAN* gene lineage in lycophytes and ferns, together with previous analyses for other genes involved in the adaxial/abaxial leaf developmental genetic network, such as *Class III HD-Zip* genes [8], allow us to better understand changes of this genetic network across the evolution of vascular plants. *Class III HD-Zip* genes are expressed on the adaxial side of leaves from across ferns with diverse leaf morphologies [8], although *KAN* expression in ferns was only assessed in one species, it was found restricted to the abaxial side of the leaves (Figure 4). Therefore, our study provides further molecular genetic support that ferns share a broad leaf developmental mechanism with seed plants [1,8].

Expression patterns of an adaxial/abaxial network in lycophytes are different, as *Class III HD-Zips* have not been detected in the adaxial side of leaves in *Selaginella moellendorffii* or other lycophytes [8,30,31], and *KANADI* homologs were not detected in the abaxial side of the leaves (Figure 3). It would appear that the lycophyte *Selaginella moellendorffii* does not use the same genetic module to specify the abaxial and adaxial identities of its leaves. However, similar to what is found in angiosperms, the expression of the *KANADI* homologs in *S. moellendorffi* is found in the phloem. This may represent the ancestral function of *KANADI* gene lineage in vascular plants.

There is a potential to unravel the evolution of the *Class III HD-Zip–KANADI* leaf developmental module by studying the expression and function of these genes throughout lycophytes and ferns. The ancestral function of *Class III HD-Zip* was proposed to be in meristem development, as these genes were found in all vascular plants, and in flowering plants are found expressed in vasculature, meristem, and the adaxial side of lateral organs [30]. However, previous expression analyses showed that *Class III HD-Zip* orthologs are not expressed in the meristems of the lycophyte *Selaginella moellendorffii* or the meristems of the eusporangiate ferns *Equisetum diffusum*, *Psilotum nudum*, or *Osmunda regalis* [8,31]. However, expression of *Class III HD-Zip* orthologs was detected in the meristem of the leptosporangiate ferns *Pilularia globulifera* and *Elaphoglossum peltatum* [8]. Here we show that *KANADI* homologs are not expressed in the meristem of the lycophyte *S. moellendorffii,* but are expressed in the meristem of the fern *Equisetum hyemale*. It will be important to further study the expression of *KANADI* homologs in lycophytes and ferns to better understand when these genes acquired meristem expression, providing more data to untangle the evolution of the *Class III HD-Zip* and *KANADI* developmental module.

Although, we did not detect the expression of *KAN* homologs in the abaxial side of developing microphylls of *Selaginella moellendorffii,* we did find that *KAN* homologs are expressed in developing sporangia (Figure 3). This is similar to the expression profile previously found for *Class III HD-Zips* with expression in sporangia [8]. This may provide support for a modified telome theory with microphylls evolving by the progressive sterilization of sporangia [3]. This result also raises an intriguing question about the molecular genetics of microphyll development of which we still know little.

## 5. Conclusions

Our results suggest that the genetic network that determines abaxial identity is conserved within ferns, gymnosperms and angiosperms but not in lycophytes. However, additional studies are necessary. Identifying the precise role of *KAN* homologs across vascular plants will require an exhaustive evaluation of spatiotemporal expression patterns throughout ferns as well as in other lycophytes, coupled with functional analyses in diverse vascular plant species.

## Supplementary Materials

The following are available online at https://www.mdpi.com/2223-7747/8/9/313/s1. Table S1: List of genes included in the ML analysis. Table S2: Primers used for expression analyses in *E. hyemale* and *S. moellendorffii*.
