# Supplementary material for: The Evolution of the KANADI Gene Family and Leaf Development in Lycophytes and Ferns
Source: Plants (Basel). 2019 Aug 30;8(9):313. doi: 10.3390/plants8090313 (PMC6783990; doi:10.3390/plants8090313)
Supplement: Supplementary file 1 [file plants-08-00313-s001.zip › supple/plants-551959-supple.pdf]

ISH primers Selaginella moellendorffii:

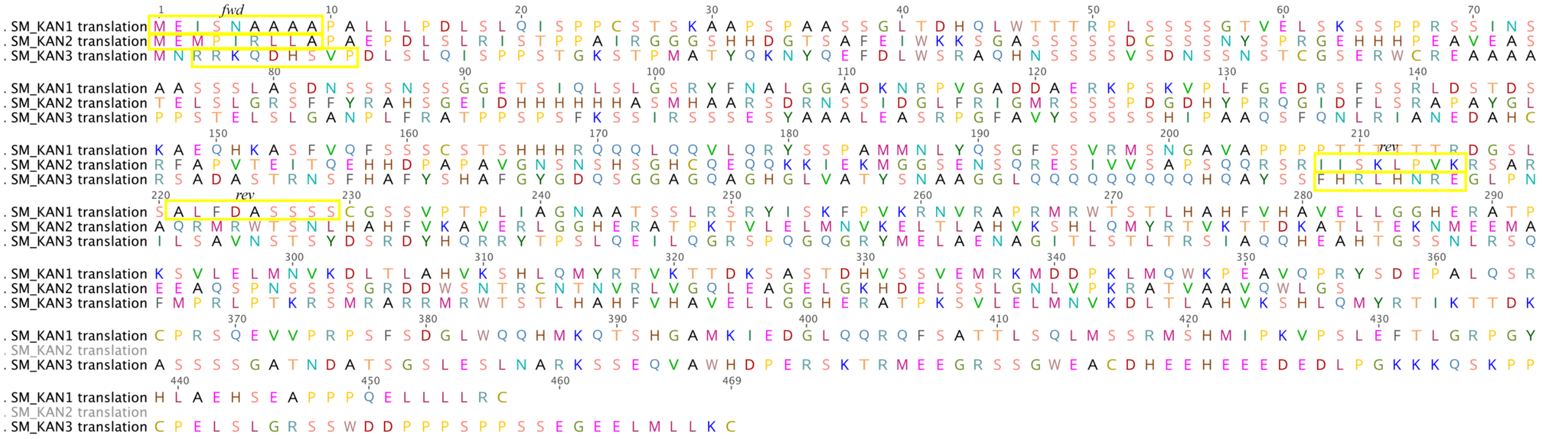

Gene	Species	Family	Accession number	Database
ArlyKAN1	*Arabidopsis lyrata* (L.) O'Kane & Al-Shehbaz	Brassicaceae	XM_021022418	NCBI
AtKAN1	*Arabidopsis thaliana* (L.) Heynh.	Brassicaceae	At5g16560	NCBI
ArhaKAN2	*Arabidopsis halleri* (L.) O'Kane & Al-Shehbaz	Brassicaceae	Araha.2690s0004.1	Phytozome
AtKAN2	*Arabidopsis thaliana* (L.) Heynh.	Brassicaceae	At1g32240	NCBI
AtKAN3	*Arabidopsis thaliana* (L.) Heynh.	Brassicaceae	At4g17695	NCBI
ArlyKAN2	*Arabidopsis lyrata* (L.) O'Kane & Al-Shehbaz	Brassicaceae	AL1G46380.t1	Phytozome
ArhaKAN3	*Arabidopsis halleri* (L.) O'Kane & Al-Shehbaz	Brassicaceae	Araha.450750021	Phytozome
ArlyKAN3	*Arabidopsis lyrata* (L.) O'Kane & Al-Shehbaz	Brassicaceae	AL7G36890.t1	Phytozome
Lybakan3	*Lycium barbarum* L.	Solanaceae	LWCK_scaffold_20128041	OneKP
LybaKAN3.2	*Lycium barbarum* L.	Solanaceae	LWCK_scaffold_20128031	OneKP
LyspKA2/3	*Lycium sp.* L.	Solanaceae	OSMU_scaffold_20775471	OneKP
SolaKAN2/3	*Solanum lasiophyllum* Humb. & Bonpl. ex Dunal	Solanaceae	DLAI_scaffold_20168201	OneKP
TsheKAN	*Tsuga heterophylla* (Raf.) Sarg.	Pinaceae	GAMH_scaffold_20502891	OneKP
ArspKAN	*Araucaria* sp. Juss.	Araucariaceae	ACWS_scaffold_20705791	OneKP
LafrKAN	*Lagarostrobos franklinii* (Hook. f.) Quinn	Podocarpaceae	ZQWM_scaffold_20857481	OneKP
JuscKAN	*Juniperus scopulorum* Sarg.	Cupressaceae	XMGP_scaffold_20580761	OneKP
FohoKAN	*Fokienia hodginsii* (Dunn) A. Henry & H.H. Thomas	Cupressaceae	UEVI_scaffold_20615841	OneKP
ChlaKAN	*Chamaecyparis lawsoniana* (A. Murray) Parl.	Cupressaceae	AIGO_scaffold_20111741	OneKP
TotaKAN	*Torreya taxifolia* Arn.	Taxaceae	EFMS_scaffold_20786011	OneKP
TabaKAN	*Taxus baccata* L.	Taxaceae	WWSS_scaffold_20062611	OneKP
AgroKAN	*Agathis robusta* (C. Moore ex F. Muell.) F.M. Bailey	Araucariaceae	MIXZ_scaffold_20107471	OneKP
AtATS	*Arabidopsis thaliana* (L.) Heynh.	Brassicaceae	At5g42630	NCBI
ArhaATS	*Arabidopsis halleri* (L.) O'Kane & Al-Shehbaz	Brassicaceae	Araha.21611s0001.1	phytozome
CisiATS	*Citrus sinensis* Pers.	Rutaceae	orange1.1g018802m	Phytozome
CiclATS	*Citrus clementina* hort.	Rutaceae	Ciclev10001679m	Phytozome
SolyATS	Solanum lycopersicum L.	Solanaceae	Solyc04g079600.2.1	Phytozome
	**Ferns**			
EdiKAN1	*Equisetum diffusum* D.Don	Equisetaceae	CAPN_2002682	OneKP

EdiKAN2	*Equisetum diffusum* D.Don	Equisetaceae	CAPN_2001900	OneKP
EdiKAN3	*Equisetum diffusum* D.Don	Equisetaceae	CAPN_2002684	OneKP
EdiKAN4	*Equisetum diffusum* D.Don	Equisetaceae	CAPN_2003191	OneKP
EdiKAN5	*Equisetum diffusum* D.Don	Equisetaceae	CAPN_2033999	OneKP
EdiKAN6	*Equisetum diffusum* D.Don	Equisetaceae	CAPN_2039666	OneKP
EhyKAN1	*Equisetum hyemale* L.	Equisetaceae	JVSC_2013610	OneKP
EhyKAN2	*Equisetum hyemale* L.	Equisetaceae	JVSZ_2131030	OneKP
EhyKAN3	*Equisetum hyemale* L.	Equisetaceae	JVSC_2014696	OneKP
PiglKAN1	*Pilularia globulifera* L.	Marsiliaceae	KIIX_2089319	OneKP
PiglKAN2	*Pilularia globulifera* L.	Marsiliaceae	KIIX_2011267	OneKP
OsmKAN	*Osmunda sp.* L.	Osmundaceae	UOMY_2081413	OneKP
ViliKAN1	*Vittaria lineata* (L.) Sm.	Pteridaceae	SKYV_2017962	OneKP
ViliKAN2	*Vittaria lineata* (L.) Sm.	Pteridaceae	SKYV_2017658	OneKP
BlspKAN1	*Blechnum spicant* (L.) Sm.	Blechnaceae	AFPO_2068439	OneKP
BlspKAN2	*Blechnum spicant* (L.) Sm.	Blechnaceae	AFPO_2073137	OneKP
BlspKAN3	*Blechnum spicant* (L.) Sm.	Blechnaceae	AFPO_2075041	OneKP
CracKAN1	*Cryptogramma acrostichoides* R.Br.	Adiantaceae	WQML_20000652	OneKP
CracKAN2	*Cryptogramma acrostichoides* R.Br.	Adiantaceae	WQML_2013575	OneKP
CerKAN1	*Ceratopteris thalictroides* (L.) Brongn.	Pteridaceae	PIVW_2013269	OneKP
CerKAN2	*Ceratopteris thalictroides* (L.) Brongn.	Pteridaceae	PIVW_2013269	OneKP
CerKAN3	*Ceratopteris thalictroides* (L.) Brongn.	Pteridaceae	PIVW_2012454	OneKP
CumaKAN	*Culcita macrocarpa* C. Presl	Culcitaceae	PNZO_2143811	OneKP
PoheKAN	*Polypodium hesperium* Maxon	Polypodiaceae	ZRAR_2034727	OneKP
PoacKAN	*Polystichum acrostichoides* (Michx.) Schott	Polypodiaceae	FQGQ_2073335	OneKP
WoscKAN	*Woodsia scopulina* D.C.Eaton	Woodsiaceae	YJJY_2075370	OneKP
CeriKAN1	*Ceratopteris richardii* Brongn.	Pteridaceae	iOHQ_Cfern/c/23/f1p123/12935	
CeriKAN2	*Ceratopteris richardii* Brongn.	Pteridaceae	iOHQ_Cfern/c/23/f1p123/10643	

SM-KAN1f	ATGGAGATTTCCAATGCGGCAGCAG
SM-KAN1r	GGAAGAGCTTGCATCAAACAAGGC
SM-KANrT7	CTAATACGACTCACTATAGGGGGAAGAGCTTGCATCAAACAAGGC
SM-KAN2f	ATGGAAATGCCCATCAGGCTC
SM-KAN2r	CTTCACGGGCAATTTGGAGATG
SM-KAN2rT7	CTAATACGACTCACTATAGGGCTTCACGGGCAATTTGGAGATG
EhyKAN1f	GAATGGTATAGAACTAGCCGG
EhyKAN1r	CGAAGACGCAGGTTATTGTGG
EhyKAN1rT7	CTAATACGACTCACTATAGGGCGAAGACGCAGGTTATTGTGG
EhyKAN2f	CTTCCACAGATCTCTGCTTAGC
EhyKAN2r	GGTAGCTTTGACAGGAATGG
EhyKAN2T7	CTAATACGACTCACTATAGGGGGTAGCTTTGACAGGAATGG
EhyKAN3fwd	catcatccatcatgagttatg
EhyKAN3rev	cttaggaaccatgggctgg
EhyKAN3revISH	CTTAATACGACTCACTATAGGGcttaggaaccatgggctgg

s
